# Humoral immunogenicity and tolerability of heterologous ChAd/BNT compared with homologous BNT/BNT and ChAd/ChAd SARS-CoV-2 vaccination in hemodialysis patients: A multicenter prospective observational study

**DOI:** 10.1007/s40620-022-01247-7

**Published:** 2022-01-27

**Authors:** Michael Haase, Paul Lesny, Mark Anderson, Gavin Cloherty, Michael Stec, Anja Haase-Fielitz, Mathias Haarhaus, Carla Santos-Araújo, Pedro Mota Veiga, Fernando Macario

**Affiliations:** 1 Diaverum Renal Care Center, Potsdam, Germany; 2 Diaverum AB, Hyllie Boulevard 39, 215 37 Malmö, Sweden; 3 Medical Faculty, Otto-Von-Guericke University Magdeburg, Leipziger Str. 44, 39120 Magdeburg, Germany; 4 Abbott Infectious Disease Research, Chicago, IL 60064-3500 USA; 5 Brandenburg Medical School Theodor Fontane, 16816 Neuruppin, Germany; 6 Faculty of Health Sciences Brandenburg, 14469 Potsdam, Germany; 7 Institute of Integrated Health Care Systems Research & Social Medicine, Otto-Von-Guericke-University Magdeburg, 39120 Magdeburg, Germany; 8 Department of Cardiology, Brandenburg Heart Center, Immanuel Hospital, 16321 Bernau, Germany; 9 Division of Renal Medicine, Department of Clinical Sciences, Intervention and Technology, Karolinska Institutet, Karolinska University Hospital, Stockholm, Sweden; 10 School of Education, Polytechnic Institute of Viseu, Viseu, Portugal; 11 NECE Research Unit in Business Sciences, University of Beira Interior, Covilhã, Portugal

**Keywords:** COVID-19, Kidney, mRNA-/vectored vaccines, SARS-CoV-2 spike IgG, Side effects

## Abstract

**Background:**

After the reports of severe adverse reactions to the AstraZeneca ChAdOx1-S-nCoV-19 vaccine, patients who had received one dose of ChAdOx1-S-nCoV-19 vaccine were recommended a second dose of Pfizer’s BNT162b2 vaccine. In hemodialysis patients, we compared the humoral immunogenicity and tolerability of homologous vaccination with ChAdOx1-nCoV-19/ChAdOx1-nCoV-19 (ChAd/ChAd) and BNT162b2/BNT162b2 (BNT/BNT) with heterologous vaccination of first dose of ChAdOx1-nCoV-19 and a second dose with BNT162b2 (ChAd/BNT).

**Methods:**

In a multicenter prospective observational study, SARS-CoV-2 spike-IgG antibody levels, Nucleocapsid-protein-IgG-antibodies, and vaccine tolerability were assessed 6 weeks after second SARS-CoV-2 vaccination in 137 hemodialysis patients and 24 immunocompetent medical personnel.

**Results:**

In COVID-19-naïve hemodialysis patients, significantly higher median SARS-CoV-2-spike IgG levels were found after ChAd/BNT (N = 16) compared to BNT/BNT (N = 100) or ChAd/ChAd (N = 10) (1744 [25th–75th percentile 276–2840] BAU/mL versus 361 [25th–75th percentile 120–936] BAU/mL; p = 0.009; 1744 [25th–75th percentile 276–2840] BAU/mL versus 100 [25th–75th percentile 41–346] BAU/mL; p = 0.017, respectively). Vaccinated, COVID-19-naïve medical personnel had median SARS-CoV-2 spike-IgG levels of 650 (25th–75th percentile 217–1402) BAU/mL and vaccinated hemodialysis patients with prior COVID-19 7047 (25th–75th percentile 685–10,794) BAU/mL (N = 11). In multivariable regression analysis, heterologous vaccination (ChAd/BNT) of COVID-19-naïve hemodialysis patients was independently associated with SARS-CoV-2 spike-IgG levels. The first dose of ChAd and the second dose of BNT after the first vaccination with ChAd (heterologous vaccination, ChAd/BNT) were associated with more frequent but manageable side effects compared with homologous BNT.

**Conclusions:**

Within the limitations of this study, heterologous vaccination with ChAd/BNT appears to induce stronger humoral immunity and more frequent but manageable side effects than homologous vaccination with BNT/BNT or with ChAd/ChAd in COVID-19-naïve hemodialysis patients.

**Graphical abstract:**

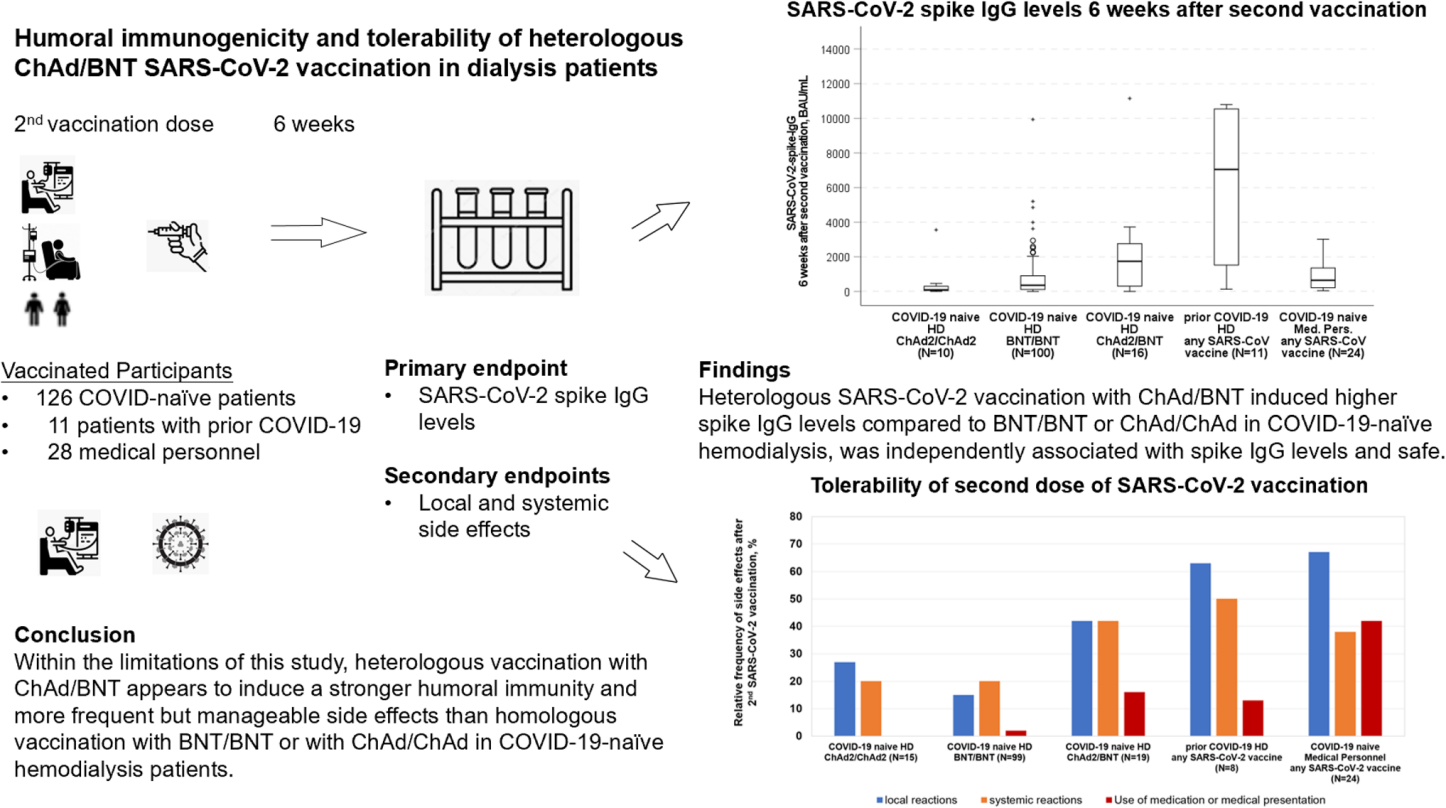

## Introduction

There is strong evidence that vaccination of the normal population with approved SARS-CoV-2-vaccines is safe and provides effective protection against SARS-CoV-2 [1, 2]. However, the optimal vaccination strategy in immunocompromised patients is still unclear [3]. In particular, hemodialysis patients generally have a high rate of vaccination failure. For example, the rate of vaccination failure for hepatitis B vaccination was 28.3% of hemodialysis patients, but < 10% in the normal population [4]. Hemodialysis patients also have a higher risk of becoming infected with COVID-19 and, when infected, have a higher associated mortality rate than the normal population [5, 6]. The immunogenicity and tolerability of SARS-CoV-2 vaccines may also differ in hemodialysis patients compared to the general population. To date, numerous studies have evaluated humoral immunity after SARS-CoV-2-vaccination in hemodialysis patients. These studies measured levels of antibodies directed against the coronavirus spike protein (SARS-CoV-2-spike-IgG). After two doses of vectored-vaccines, such as ChAdOx1-nCoV-19(AZD1222)/Oxford-AstraZeneca (ChAd/ChAd) lower SARS-CoV-2-spike-IgG levels were observed compared with levels after two doses of mRNA-vaccines such as BNT162b2/Pfizer-BioNTech (BNT/BNT) [7–11]. Recently, following rare but severe thromboembolic events in young people to the AstraZeneca ChAdOx1-S-nCoV-19 vaccine, patients who received one dose of ChAdOx1-S-nCoV-19 vaccine were recommended by European Health Authorities to receive a second dose of Pfizer’s BNT162b2 vaccine (ChAd/BNT). Accordingly, several studies have reported conflicting data regarding the immunogenicity and efficacy of heterologous ChAd/BNT compared with homologous BNT/BNT vaccination in the general population [12–15]. Despite slight differences in the tolerability favoring homologous or heterologous vaccination regimens, recent studies have concluded that heterologous prime-boost immunization strategies for COVID-19 might be generally effective in the general population. However, their effect on the humoral response in hemodialysis patients remains unmeasured. In COVID-19-naïve hemodialysis patients, we aimed to compare the humoral immunogenicity and tolerability of heterologous ChAd/BNT vaccination with that of homologous ChAd/ChAd and BNT/BNT vaccinations.

## Patients and methods

### Design, setting & participants

In a multicenter prospective observational study (02/2021–08/2021) with a follow-up period through 12/2021, we screened all hemodialysis patients treated at kidney care centers in Potsdam, Ludwigsfelde and Rangsdorf, Germany, and medical personnel, for eligibility to participate. COVID-19-naïve hemodialysis patients and patients having presented with PCR-positive-COVID-19 > 3 months before study start, as well as  immunocompetent medical personnel were enrolled after providing written informed consent for study participation and publication. Exclusion criteria were missing written informed consent, age < 18 years, and no second dose of SARS-CoV-2-vaccination before sampling for antibody-measurement. Following the prioritization by the National Vaccination Committee, all participants were vaccinated, upon availability, with either two doses of the ChAdOx1-nCoV-19(AZD1222)/Oxford-AstraZeneca (ChAd/ChAd) with dosing interval of 42–84 days; two doses of the Pfizer/BioNTech-mRNA-BNT162b2-SARS-CoV-2-vaccine (BNT/BNT) with dosing interval of 21–28 days, or a combination of one dose of ChAd followed by one dose of BNT, respectively. Specifically, the choice of performing the heterologous or homologous vaccination regimen relied on three factors: First, prioritization by the National Vaccination Committee to prefer an mRNA COVID-19 vaccine as second dose after first dose of ChAdOx1-nCoV-19(AZD1222)/Oxford-AstraZeneca in patients < 60 years. Second, availability of mRNA vaccine (BNT/Comirnaty/Biontech or mRNA1273/Spikevax/Moderna). Third, participant’s choice for receiving an mRNA vaccine as second dose or, receiving ChAd as second dose.

The procedures used in this study adhere to tenets of the Declaration of Helsinki. Study approval was obtained from the Ethics Committee of ‘*Landesärztekammer Brandenburg’*, Germany (S9/(bB)/2021). This manuscript adheres to the ‘*Strengthening the Reporting of Observational Studies in Epidemiology*’ guidelines [16].

### Endpoints

The primary endpoint was SARS-CoV-2-spike-IgG-level 6 weeks after the second vaccine dose in COVID-19-naïve hemodialysis patients with ChAd/ChAd or BNT/BNT compared with those with ChAd/BNT. Secondary endpoints were the proportion of patients with local or systemic side effects after the first or second vaccination, respectively; and the proportion of patients with medication use or concurrent medical presentation within the first week after first or second SARS-CoV-2 vaccination, respectively.

### SARS-CoV-2-antibody measurement

Serum of participants was collected immediately before a first SARS-CoV-2 vaccination, at 2 weeks after receiving a first and at 6 weeks after a second vaccine dose. Participants were tested for SARS-CoV-2-IgG-antibody levels from serum directed against the spike protein (SARS-CoV-2-spike-IgG), and IgG-antibodies directed against the Nucleocapsid protein [17]. All samples were run on Abbott ARCHITECT™ i2000SR instrument (Abbott Park, IL). The FDA- EUA-approved SARS-CoV-2-IgG (List 6R86), and SARS-CoV-2-IgG-II Quant (List 6S60) assays were used, both automated Chemiluminescent Microparticle Immunoassays (CMIA). Assay results are reported as an index value of the ratio of specimen to calibrator Relative Light Units (RLU) signal. The SARS-CoV-2-IgG-II-Quant-assay is an automated CMIA used for quantitative detection of IgG-antibodies (BAU/mL) directed against the receptor-binding-domain of the SARS-CoV-2-spike protein (assay linearity 3–5680 BAU/mL). The laboratory investigators were blinded to sample sources and clinical outcomes. Researchers who obtained and assessed clinical data were blinded to antibody-measurements.

### SARS-CoV-2-vaccination tolerability questionnaire

Vaccination side effects in study participants were evaluated in accordance with the pivotal study of Polack et al. [1]. Information about the occurrence of local and systemic side effects, and medication use or concurrent medical presentation after the first and second vaccination was obtained in face-to-face interviews with study participants using prepared questionnaires during the first week after vaccination. Patients filled in questionnaires independently or with the help of a nurse.

### Statistical analysis

For evaluation of the immunogenicity and tolerability of SARS-CoV-2 vaccines, study size was determined by date of serum sampling or obtaining the tolerability questionnaire before 20th July, 2021 to gather early potentially important clinical information for this patient population. Linear values in the text, tables and figures are presented as median (25th–75th percentile) unless specified otherwise.

According to the study hypothesis, SARS-CoV-2-IgG-related humoral immunogenicity and tolerability of heterologous vaccination with ChAd/BNT and homologous vaccinations with ChAd/ChAd or BNT/BNT in COVID-19-naïve hemodialysis patients was analyzed. To control the type 1 error rate, we used the closed test procedure as suggested by Markus et al. [18] and Bauer [19].

Accordingly, first, three-group comparison by Kruskal–Wallis-test or Chi^2^/Fisher’s exact test of trend for all single hypotheses was performed, and, if a significant effect was found, pairwise comparison by Mann–Whitney-U-test or Chi^2^/Fisher’s exact test was done. Independent risk factors for log SARS-CoV-2-spike-IgG-levels 6 weeks after the second vaccination in hemodialysis patients were identified by including vaccination type (ChAd/ChAd, BNT/BNT, ChAd/BNT) and clinically relevant variables previously identified in the literature [7–11] or those with univariate p < 0.2 into a multivariable linear regression analysis that included sex and log-values of age, Charlson-Comorbidity-Index, dialysis vintage, serum albumin levels and anti-hepatitis B surface (HBs)-antibody levels. Immunogenicity and tolerability of SARS-CoV-2-vaccination in patients with prior COVID-19, and for medical personnel were reported for descriptive purpose and to facilitate data interpretation. Alpha was set at 0.05 (2-tailed). SPSS, version 26.0 (IBM Corp., Armonk, NY, USA) was used.

## Results

### Participant characteristics

**Fig. 1:**
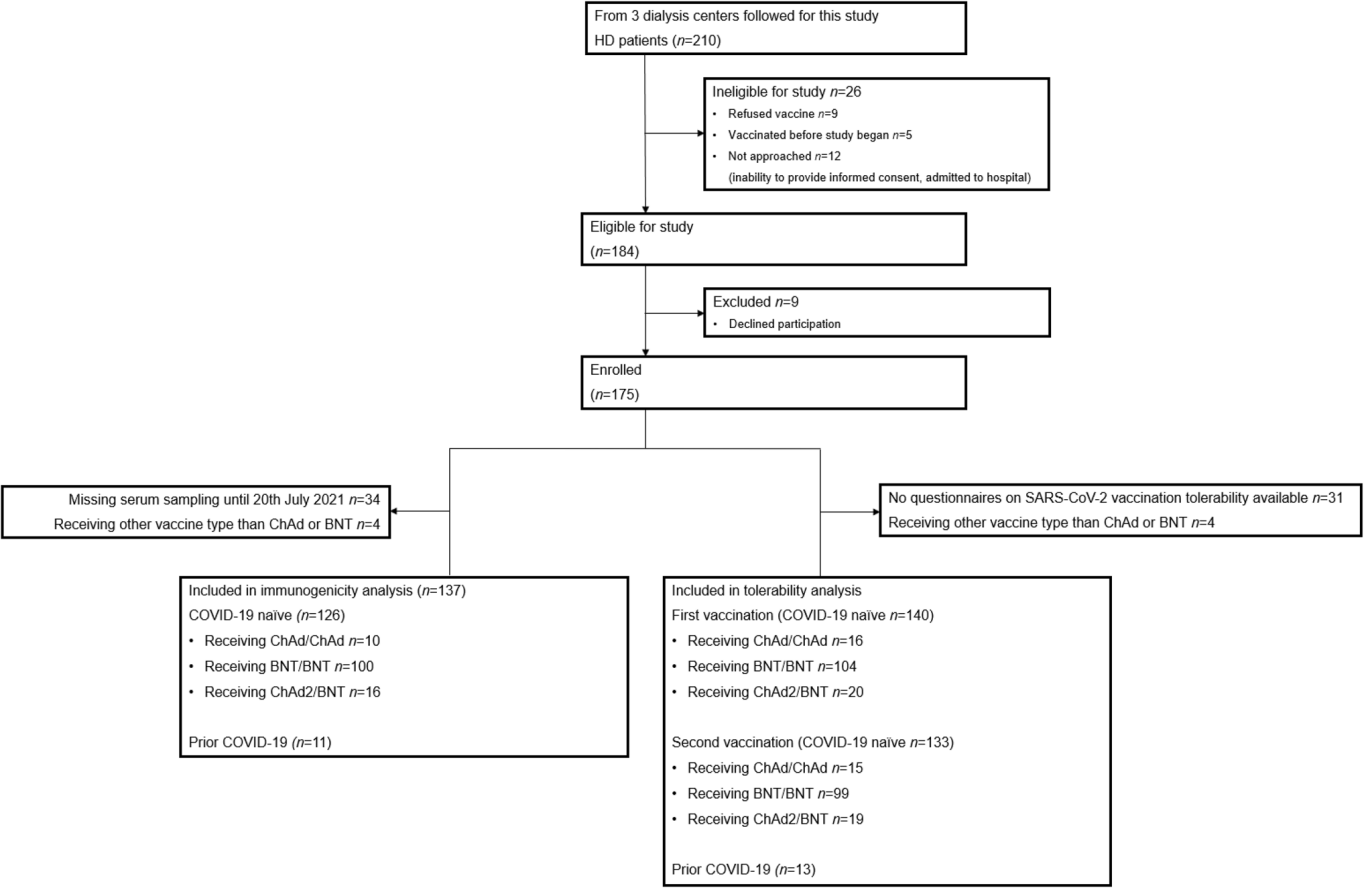
Flow of patients through the study

Of 210 hemodialysis patients, 175 were enrolled in the study. The SARS-CoV-2-spike-IgG-levels of 137 patients with two doses of SARS-CoV-2-vaccination were analyzed, including 126 COVID-19-naïve hemodialysis patients and 11 vaccinated patients with prior COVID-19 (Fig. 1). Of 126 COVID-19-naïve patients, 10 received homologous vaccination with ChAd/ChAd, 100 homologous vaccination with BNT/BNT, and 16 heterologous vaccination with ChAd/BNT. COVID-19-naïve patients represented a typical hemodialysis cohort, comprising 51% of patients with diabetes and 91% with arterial hypertension. Median patient age was 76 (25th–75th percentile 64–82) years, Charlson-Comorbidity-Index 7 (25th–75th percentile 5–8) points and dialysis vintage 45 (25th–75th percentile 18–90) months. Primary kidney disease was mainly diabetic or hypertensive nephropathy and glomerulonephritis, as shown in Table 1. In COVID-19-naïve hemodialysis patients, the median interval between first and second SARS-CoV-2-vaccination was 42 (25th–75th percentile 28–42) days and 43 (25th–75th percentile 41–43) days between second SARS-CoV-2-vaccination and sampling.

**Table 1:** Patient characteristics

	COVID-19-naïve HD ChAd/ChAd (N = 10)	COVID-19-naïve HD BNT/BNT (N = 100)	COVID-19-naïve—HD ChAd/BNT (N = 16)	P
Demographic data				
Age, years	61 (58–62)	78 (69–83)	56 (45–60)	< 0.001
Sex, m	8 (80%)	63 (63%)	14 (88%)	0.10
Body mass index, kg/m^2^	25.9 (12.0–27.0)	27.9 (24.6–33.1)	30.1 (24.9–34.2)	0.05
Charlson Comorbidity Index, points	4.0 (3.8–6.0)	7.0 (6.0–9.0)	4.0 (3.0–6.8)	< 0.001
Dialysis				
Hemodialysis vintage, months	88 (47–175)	43 (17–77)	51 (24–119)	0.08
Dialysis modality, hemodialysis/hemodiafiltration, n	5/5	78/22	10/6	0.27
Arteriovenous fistula, n	8 (80%)	80 (80%)	10 (63%)	0.14
Shunt graft, n	2 (20%)	7 (7%)	4 (25%)	
Atrial catheter, n	0 (0%)	11 (11%)	2 (13%)	
Kt/V	2.0 (1.7–2.1)	1.7 (1.5–1.9)	1.6 (1.6–1.8)	0.07
Primary kidney disease				
Diabetic nephropathy, n	1 (10%)	29 (28%)	1 (6%)	0.43
Hypertensive nephropathy, n	1 (10%)	25 (25%)	2 (13%)	
Glomerulonephritis, n	3 (30%)	15 (15%)	5 (31%)	
Other primary kidney disease, n	2 (20%)	17 (17%)	5 (31%)	
Unknown primary kidney disease, n	3 (30%)	14 (14%)	3 (13%)	
Comorbidities				
Diabetes mellitus, n	2 (20%)	57 (57%)	5 (31%)	0.020
Arterial hypertension, n	8 (80%)	92 (92%)	15 (94%)	0.53
Ischemic heart disease, n	1 (10%)	29 (29%)	4 (25%)	0.67
Peripheral artery occlusive disease, n	0 (0%)	15 (15%)	3 (19%)	0.64
Stroke, n	1 (10%)	13 (13%)	1 (6%)	0.88
Chronic obstructive pulmonary disease, n	2 (20%)	12 (12%)	3 (19%)	0.85
Malignancy, n	1 (10%)	30 (30%)	3 (19%)	0.53
Drugs				
Aspirin, n	2 (10%)	47 (47%)	8 (50%)	0.47
Statins, n	6 (60%)	51 (51%)	6 (38%)	0.72
Angiotensin receptor blockers, n	4 (40%)	47 (47%)	8 (50%)	0.87
ACE inhibitors, n	2 (20%)	25 (25%)	5 (31%)	0.92
Betablockers, n	8 (80%)	70 (70%)	13 (81%)	0.83
Calcium channel blockers, n	4 (40%)	53 (53%)	10 (63%)	0.77
Diuretics, n	7 (70%)	80 (80%)	12 (75%)	0.81
Laboratory values (prior to 1st SARS-CoV-2 vaccine.)				
Albumin (calculated), g/L	44.6 (40.6–51.0)	43.2 (40.2–47.3)	43.2 (39.6–46.8)	0.59
Hemoglobin, g/dL	11.4 (10.3–11.7)	10.9 (10.0–11.4)	11.4 (10.3–12.4)	0.25
Ferritin, mg/dL	413 (285–445)	458 (323–578)	428 (272–593)	0.93
Anti HBs-levels, mIU/mL	306 (17–1,000)	190 (1–818)	92 (1–445)	0.59

Numbers denote median (25th–75th percentile)

Vaccinated patients with prior COVID-19 included three males. They were aged median 78 (25th–75th percentile 64–82) years, had a median BMI of 27.5 (25th–75th percentile 25.1–28.2), a median Charlson Comorbidity Index of 6.0 (25th–75th percentile 6.0–9.0) points and a median hemodialysis vintage of 80 (25th–75th percentile 40–128) months. Among them, one received the ChAd vaccine, eight BNT vaccine and two heterologous vaccinations with ChAd/BNT.

Of thirty-one vaccinated medical personnel, three had prior COVID-19. Twenty-eight vaccinated COVID-19-naïve medical personnel were analyzed (20 females, 8 males). Among them, ten received two doses of ChAd, eight two doses of BNT and ten one dose of ChAd followed by one dose of BNT. Median age of vaccinated COVID-19-naïve medical personnel was 45 (25th–75th percentile 37–56) years. The median interval between second SARS-CoV-2-vaccination and sampling was 43 (25th–75th percentile 42–46) days.

No individual Nucleocapsid-IgG-result was > 1.4 Index for positive test indicating no previously undetected COVID-19 infection in any COVID-19-naïve participant. Since COVID-19 vaccinations started being administered at our study centers, we observed three COVID-19 breakthrough infections in initially COVID-19 naïve, vaccinated hemodialysis patients and one in initially COVID-19 naïve, medical personnel (homologous ChAd/ChAd). Of these three patients with COVID-19 breakthrough infections, one received homologous BNT/BNT and survived COVID-19, one homologous BNT/BNT and did not survive COVID-19 and one homologous ChAd/ChAd surviving COVID-19 after receiving COVID-19 antibodies early in the course.

During participant follow-up, overall, no medical personnel, but twelve hemodialysis patients died (3/10 [30%] homologous ChAd/ChAd, 8/100 [8%] homologous BNT/BNT, and 1/16 [6%] heterologous ChAd/BNT).

### Humoral immunogenicity of SARS-CoV-2 vaccination

**Fig. 2:**
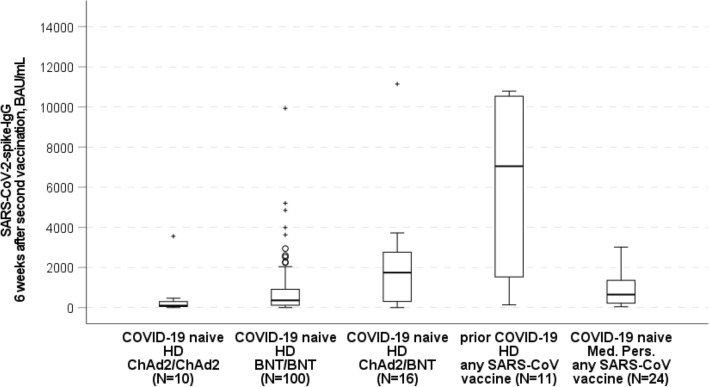
SARS-CoV-2-spike-IgG levels 6 weeks after second vaccination (patients with vaccination after prior COVID-19 infection and COVID-naïve, vaccinated medical personnel were reported for descriptive purpose). 3-group comparison (Kruskal–Wallis test): p = 0.006, 2-group comparisons (Mann–Whitney *U* test): ChAd/ChAd vs. BNT/BNT: p = 0.07,BNT/BNT vs. ChAd/BNT: p = 0.009,ChAd/ChAd vs. ChAd/BNT: p = 0.017,ChAd/ChAd, homologous vaccination with two doses of ChAdOx1-nCoV-19(AZD1222)/Oxford-AstraZeneca. BNT/BNT, homologous vaccination with two doses of BNT162b2/Pfizer-BioNTech. ChAd/BNT, heterologous vaccination with one dose of ChAdOx1-nCoV-19(AZD1222)/Oxford-AstraZeneca followed by one dose of BNT162b2/Pfizer-BioNTech. *HD* hemodialysis patients

SARS-CoV-2-spike-IgG-levels and Nucleocapsid-IgG-levels before, 2 weeks after first and 6 weeks after second vaccination are shown in Table 2. Six weeks after the second vaccination, there was a significant difference in the primary endpoint, SARS-CoV-2-spike-IgG-levels, comparing ChAd/ChAd, BNT/BNT and ChAd/BNT in COVID-19-naïve patients (Kruskal–Wallis-test, p = 0.006). This difference resulted from significantly higher SARS-CoV-2-spike-IgG-levels in patients receiving heterologous vaccination with ChAd/BNT at median 1744 (25th–75th percentile 276–2840) BAU/mL compared to homologous vaccination with BNT/BNT at median 361 (25th–75th percentile 120–936) BAU/mL, p = 0.009, and homologous vaccination with ChAd/ChAd at median 100 (25th–75th percentile 41–346) BAU/mL, p = 0.017 (Fig. 2).

**Table 2:** COVID-19-naïve hemodialysis patients – SARS-CoV-2 antibody levels before and after 1^st^ or 2^nd^ SARS-CoV-2 vaccination

	COVID-19-naïve HD patients ChAd/ChAd (N = 10)			COVID-19-naïve HD patients BNT/BNT (N = 100)			COVID-19-naïve HD patients ChAd/BNT (N = 16)		
	Before 1st vaccin.	2 weeks after 1st vaccin.	6 weeks after 2nd vaccin.	Before 1st vaccin.	2 weeks after 1st vaccin.	6 weeks after 2nd vaccin.	Before 1st vaccin.	2 weeks after 1st vaccin.	6 weeks after 2nd vaccin.
IgG spike, BAU/mL^a^	0.1 (0.0–0.3)	0.9 (0.3–5.4)	100 (41–346)	0 (0.0–0.3)	4 (0.4–18.7)	361 (120–936)	0 (0–0.4)	1.9 (1.7–3.4)	1744 (276–2840)
IgG Nucleocapsid, Index^b^	0.1 (0.0–0.2)	0.0 (0.0–0.2)	0.0 (0.0–0.3)	0.0 (0.0–0.1)	0.0 (0.0–0.1)	0.0 (0.0–0.1)	0.0 (0.0–0.1)	0.0 (0.0–0.2)	0.0 (0.0–0.1)

Numbers denote median (25th–75th percentile)

^a^Test positive, if > 7 BAU/mL, means

^b^Test positive, if Index > 1.4

Multivariable regression analysis showed that, in COVID-19-naïve hemodialysis patients, heterologous vaccination and anti-HBs antibodies before SARS-CoV-2 vaccination were independent predictors of SARS-CoV-2-spike-IgG-levels 6 weeks after second vaccination (Table 3), whereas previously identified modifiers including age, sex, Charlson-Comorbidity-Index, diabetes, dialysis vintage, and serum albumin-levels, were not.

**Table 3:** Predictors of SARS-CoV-2 spike IgG levels 6 weeks after second vaccination of COVID-19**-naïve** hemodialysis patients

Variable	Adjusted B coefficient (95% CI)	*P*
Age^a^, years	– 1.15 (– 3.53 to 1.22)	0.336
Sex	– 0.06 (– 0.43 to 0.32)	0.759
Diabetes mellitus	0.14 (– 0.24 to 0.53)	0.464
Charlson Comorbidity Index^a^, points	0.34 (– 1.24 to 1.91)	0.673
Dialysis vintage^a^, months	– 0.37 (– 0.78 to 0.04)	0.077
Serum albumin levels^a^, g/L	2.39 (– 0.70 to 5.48)	0.128
Hepatitis B antibody levels (anti-HBs)^a^, mIU/mL	0.27 (0.12 to 0.41)	0.001
Vaccination type^b^	– 0.58 (– 1.12 to – 0.05)	0.032

^a^For non-normally distributed parameters, log values were used

^b^Homologous SARS-CoV-2 vaccination with ChAd/ChAd or BNT/BNT versus heterologous SARS-CoV-2 vaccination with ChAd/BNT

In addition, to facilitate interpretation of the results in COVID-19-naïve hemodialysis patients, we assessed the humoral immunogenicity associated with SARS-CoV-2 vaccination for hemodialysis patients with prior COVID-19 and COVID-19-naïve medical personnel 6 weeks after second vaccination (Fig. 2). Medical personnel showed median SARS-CoV-2-spike-IgG-levels of 650 (25th–75th percentile 217–1402) BAU/mL. However, numerically higher SARS-CoV-2-spike-IgG-levels were found in vaccinated hemodialysis patients with prior COVID-19 (median 7047 [25th–75th percentile 685–10,794] BAU/mL).

### Tolerability of SARS-CoV-2 vaccination

Of 175 patients enrolled, questionnaires on tolerability of first SARS-CoV-2 vaccination were available from 140 COVID-19-naïve hemodialysis patients and 27 medical personnel, and, of second vaccination from 133 COVID-19-naïve hemodialysis patients and 24 medical personnel. Also, questionnaires on tolerability of first SARS-CoV-2 vaccination were available from 13 vaccinated hemodialysis patients with prior COVID-19.

**Fig. 3:**
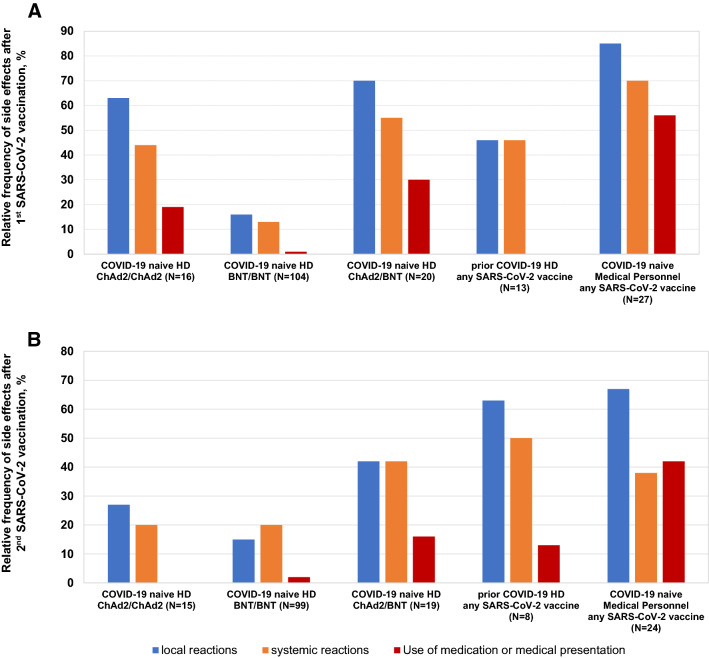
Tolerability of SARS-CoV-2 vaccination. **a** First dose. *Local side effects***.** 3-group comparison (Kruskal–Wallis test): p < 0.001. 2-group comparisons (Mann–Whitney *U* test): BNT/BNT vs. ChAd/BNT: p < 0.001, BNT/BNT vs. ChAd/ChAd: p < 0.001, ChAd/ChAd vs. ChAd/BNT: p = 0.635. *Systemic side effects *3-group comparison (Kruskal–Wallis test): p < 0.001. 2-group comparisons (Mann–Whitney *U* test): BNT/BNT vs. ChAd/BNT: p < 0.001, BNT/BNT vs. ChAd/ChAd: p = 0.002, ChAd/ChAd vs. ChAd/BNT: p = 0.502. *Medication use or medical presentation* 3-group comparison (Kruskal–Wallis test): p < 0.001, 2-group comparisons (Mann–Whitney *U* test): BNT/BNT vs. ChAd/BNT: p < 0.001, BNT/BNT vs. ChAd/ChAd: p = 0.007, ChAd/ChAd vs. ChAd/BNT: p = 0.700*.* **b **Second dose. *Local side effects *3-group comparison (Kruskal–Wallis test): p = 0.023. 2-group comparisons (Mann–Whitney *U* test): BNT/BNT vs. ChAd/BNT: p = 0.007, BNT/BNT vs. ChAd/ChAd: p = 0.273, ChAd/ChAd vs. ChAd/BNT: p = 0.478. *Systemic side effects *3-group comparison (Kruskal–Wallis test): p = 0.112 (no two-group comparisons were performed). *Medication use or concurrent medical presentation* 3-group comparison (Kruskal–Wallis test): p = 0.011, 2-group comparisons (Mann–Whitney *U* test): BNT/BNT vs. ChAd/BNT: p = 0.029, BNT/BNT vs. ChAd/ChAd: p = 0.99, ChAd/ChAd vs. ChAd/BNT: p = 0.238. ChAd/ChAd, homologous vaccination with two doses of ChAdOx1-nCoV-19(AZD1222)/Oxford-AstraZeneca, BNT/BNT, homologous vaccination with two doses of BNT162b2/Pfizer-BioNTech, ChAd/BNT, heterologous vaccination with one dose of ChAdOx1-nCoV-19(AZD1222)/Oxford-AstraZeneca followed by one dose of BNT162b2/Pfizer-BioNTech. *HD* hemodialysis patients

Of 140 COVID-19-naïve patients after first vaccination, 16 received ChAd (with ChAd as second vaccination), 104 BNT and 20 ChAd (with BNT as second vaccination). Differences in local and systemic reactions and medication use or concurrent medical presentation following the first SARS-CoV-2 vaccination among COVID-19-naïve patients were observed (Kruskal–Wallis-test, all p < 0.001) (Fig. 3a). Such difference resulted from fewer side effects after BNT compared to ChAd (Mann–Whitney-*U*-test all p < 0.01).

Of 133 COVID-19-naïve patients with second vaccination, 15 received ChAd/ChAd, 99 BNT/BNT and 19 ChAd/BNT. Differences in local (but not systemic) reactions and medication use or concurrent medical presentation following the second SARS-CoV-2 vaccination among COVID-19-naïve patients were observed (Kruskal–Wallis-test, p < 0.05) (Fig. 3b). Such differences resulted from fewer side effects after BNT compared to ChAd (Mann–Whitney-*U*-test p < 0.05).

Median duration of symptoms after first dose of ChAd or BNT were 2 days (25th–75th percentile 1–3), 1 day (25th–75th percentile 1–2) and 1 day (25th–75th percentile 1–2), respectively. Median duration of symptoms after second dose of homologous ChAd/ChAd, BNT/BNT or ChAd/BNT were 1 day (25th–75th percentile 1–1), 1 day (25th–75th percentile 1–2) and 1 day (25th–75th percentile 1–2), respectively.

Side effects to vaccinations of COVID-19-naïve medical personnel and hemodialysis patients with prior COVID-19 are shown in Fig. 3a, b.

## Discussion

Recently, potential immunologic and logistic advantages of heterologous versus homologous SARS-CoV-2 vaccination in the normal population have been reported. Accordingly, we conducted a multicenter prospective observational study to evaluate the humoral immunogenicity and tolerability of homologous ChAdOX1 (ChAd/ChAd) and BNT162b2 (BNT/BNT) vaccinations with that of heterologous ChAdOX1/BNT162b2 (ChAd/BNT) vaccination in a hemodialysis patient cohort.

First, we found that heterologous ChAd/BNT vaccination elicited significantly higher immunity in COVID-19-naïve hemodialysis patients compared with homologous ChAdOX1 (ChAd/ChAd) and BNT162b2 (BNT/BNT) vaccination, (ChAd/BNT vs. ChAd/ChAd, antibody titer ratio: 17.5; ChAd/BNT vs. BNT/BNT, antibody titer ratio: 4.8). These results remained significant after adjustment for important covariates. Second, importantly, the safety profile of heterologous and homologous SARS-CoV-2 vaccinations was characterized by short-term, mild-to-moderate side effects in patients and medical personnel. Heterologous SARS-CoV-2-vaccination in COVID-19-naïve hemodialysis patients resulted in more frequent, but manageable side effects. Third, heterologous SARS-CoV-2 vaccination induced a numerically stronger humoral response in COVID-19-naïve hemodialysis patients than in vaccinated COVID-19-naïve medical personnel. Finally, the numerically highest SARS-CoV-2-spike-IgG-levels were seen in vaccinated hemodialysis patients with prior COVID-19.

Hemodialysis patients are considered immunocompromised and substantial rates of non-responder to SARS-CoV-2-vaccinations have been demonstrated [7]. Only a few studies, however, provided SARS-CoV-2-spike-IgG-levels from a control group, making the interpretation of their findings quite complex. In addition, decreasing antibody levels were observed in dialysis patients [20]. In the normal population, several studies reported robust to higher immunogenicity of heterologous ChAd/BNT vaccination compared to that of homologous BNT/BNT [14] or ChAd/ChAd [12, 13, 21] vaccinations. The type of repeated vaccinations to increase immunity, however, may be different in hemodialysis patients.

Regarding hesitancy of hemodialysis patients regarding SARS-CoV-2-vaccines, Garcia et al. showed that one in five were reluctant to seek the COVID-19 vaccine even if the vaccine was considered safe for the general population [22]. A recent study from France and Italy showed that concerns about side effects and vaccine efficacy in dialysis patients were independent predictors for higher vaccine hesitancy [23].

To date, no study has reported on the immunogenicity and tolerability of heterologous versus homologous SARS-CoV-2 vaccination in hemodialysis patients.

The present study findings are novel,  and report on differences in the immunogenicity and potential side effects of heterologous compared to homologous SARS-CoV-2 vaccination in hemodialysis patients.

There are several potential explanations for the observed increased humoral immunogenicity of heterologous vaccination, as evidenced by higher spike-IgG-levels in hemodialysis patients after heterologous compared with homologous vaccination. First, the patients who received heterologous vaccination in the present study were the youngest and they suffered less frequently from diabetes, both of which are factors that may favor increased humoral immunity and SARS-CoV-2 spike IgG levels. However, heterologous vaccination remained an independent predictor of humoral immunity after adjustment for age and diabetes status. In addition, first vaccination with ChAd induced numerically similar SARS-CoV-2 spike IgG levels in patients receiving BNT as second vaccination compared to homologous BNT/BNT or ChAd/ChAd vaccinations. Second, although a closed test procedure was used, it cannot be ruled out that the main finding of the present study is a random result or that unknown factors may have contributed to increased immunity after heterologous vaccination. However, the use of two different homologous SARS-CoV-2 vaccinations (ChAd/ChAd and BNT/BNT) and vaccinated hemodialysis patients with prior COVID-19 and COVID-19-naïve medical personnel as control groups—both showing higher IgG levels compared to dialysis patients receiving BNT/BNT or ChAd/ChAd as reported in other studies [24, 25]—reduces the likelihood of a chance finding of increased immunogenicity after heterologous vaccination in hemodialysis patients. Third, the following observations support the biological plausibility of the present study findings. The pattern of markedly increased vaccine immunogenicity and moderately decreased tolerability of heterologous ChAd/BNT vaccination compared with homologous vaccinations in COVID-19-naïve hemodialysis patients now demonstrated is consistent with observations of four recent studies in the normal population [12–14, 21]. There is evidence that, in the normal population, immunogenicity and efficacy advantages and tolerability disadvantages of heterologous versus homologous vaccination may be due to the additive effects of vectored-vaccines rather activating T-cell function and mRNA-vaccines stimulating IgG-antibody response [14, 26] possibly explaining, in part, present study findings. The SARS-CoV-2 vaccine also consolidates antibody immunity in infected and surviving hemodialysis patients, which has also been recently described [27]. The finding of highest SARS-CoV-2-spike-IgG-levels in vaccinated hemodialysis patients with prior COVID-19 is also consistent in magnitude with SARS-CoV-2-spike-IgG-levels reported for the vaccinated normal population with prior COVID-19 [28].

Nonetheless, the higher SARS-CoV-2 spike IgG levels in hemodialysis patients receiving heterologous vaccination must be cautiously interpreted and motivate subsequent prospective studies.

In the literature, there is conflicting data regarding the tolerability of homologous compared to heterologous SARS-CoV-2 vaccination regimens in the general population. While two studies observed fewer and less severe vaccination side effects of heterologous vaccinations [12, 14], the interim results of the ‘*Com-COV’ trial* reported increased systemic vaccine reactions after heterologous ChAd-BNT vaccination compared with homologous ChAd and BNT schedules in the general population [15]. In a recent study, hemodialysis patients appeared to develop fewer and less severe vaccination side effects compared to immunocompetent medical personnel after SARS-CoV-2 vaccinations [29], but the tolerability of homologous or heterologous vaccination regimens remained unclear in this patient population. We speculate that the observed higher reactogenicity of heterologous compared to homologous vaccination in the present study may be related to activation of different humoral and cellular immune pathways through different types of vaccine such as mRNA- and vector-based agents.

The results of the present study suggest that heterologous ChAd/BNT vaccination may be preferable as the primary choice in hemodialysis patients where both vaccines are available. Although the observed mild-to-moderate vaccination side effects were transient, they must be considered when using a heterologous vaccination schedule. Yet, the findings of our study imply that any reluctance to receive combinations of vaccines for fear of serious side effects is not justified. The results of our study demonstrating immunogenic and safe immunization against SARS-CoV-2 in hemodialysis patients using heterologous ChAd/BNT vaccination could help optimize logistics, improve immunogenicity, and mitigate potential shortages in the supply of individual vaccines, not only in Europe.

We suggest that COVID-19-naïve hemodialysis patients should be prioritized for a third dose, particularly patients who have received homologous ChAd/ChAd vaccination. This suggestion is also supported by a recent report on hemodialysis patients, in which homologous ChAd/ChAd vaccination induced lower SARS-CoV-2-spike-IgG-levels in COVID-19-naïve hemodialysis patients than homologous BNT/BNT vaccination [30]. Also, in a recent study of hemodialysis patients, a third dose of BNT162b2 allowed seroconversion in more than half of non-responders [31] or enhanced humoral response in almost all hemodialysis patients. Dialysis patients with low humoral response may benefit from a third dose of BNT162b2 mRNA COVID-19 vaccine [24].

The study has some limitations. The allocation of vaccination combination was not random, thereby creating the potential for selection bias. However, a stronger humoral response to a vaccine is not necessarily associated with more effective COVID-19 prevention. Nonetheless, in the general population, before improved efficacy was demonstrated for heterologous ChAd/BNT vaccination [26], a more pronounced humoral, spike-related immunity was shown [12–14]. In the present study, cellular immunity was not measured. Such measurement as well as measurement of decreasing antibody levels over time would be needed to improve the understanding of immunity of dialysis patients after SARS-CoV-2 vaccinations.

Our study also has several strengths. The size of the cohort was large enough to allow us identifying differences. The patients represented a typical population of patients on dialysis in a high income country. The differences observed were clear and consistent. The assessment was blinded to vaccination characteristics. A control population of health care workers provided an additional perspective. The report of limited side effects is important in decreasing vaccine hesitancy.

### Conclusion

In conclusion, within the limitations of this study, heterologous vaccination with ChAd/BNT appears to induce stronger humoral immunity in COVID-19-naïve hemodialysis patients than homologous vaccination with ChAd/ChAd or BNT/BNT. More, but manageable side effects occurred with heterologous vaccination compared with homologous vaccination. This information can be used to assist both clinicians and patients in their choice of preferred booster vaccination.

## Data Availability

The datasets generated and/or analyzed during the current study are available from the corresponding author on reasonable request.
